# Symmetric hyperkeratotic plaques of palms and soles: a case of palmoplantar psoriasis

**DOI:** 10.11604/pamj.2025.52.123.49030

**Published:** 2025-11-21

**Authors:** Kamlesh Surendra Choudhary, Anita Santoshrao Wanjari

**Affiliations:** 1 Department of Rasashastra Evam Bhaishajya Kalpana, Mahatma Gandhi Ayurveda Collage Hospital and Research Center, Datta Meghe Institute of Higher Education and Research, Salod (H) Wardha, Maharashtra, India

**Keywords:** Keratoderma, psoriasis, erythematous plaques

## Image in medicine

A 56-year-old female agricultural worker from a tropical region presented with progressive thickening and painful callus-like lesions on both palms and soles over the past six months. She reported no history of trauma or systemic illness. Initially, she used over-the-counter moisturizers and emollients, which offered minimal relief. Over time, the lesions worsened, leading to painful fissures, a burning sensation, and difficulty performing manual work and walking. A course of topical antifungals and steroids provided no lasting improvement. On clinical examination, diffuse hyperkeratotic plaques with whitish to blackish discoloration, scaling, and deep fissures were noted symmetrically on both palms and soles. Based on history and findings, a diagnosis of palmoplantar psoriasis was made. Palmoplantar psoriasis is a chronic inflammatory skin disorder characterized by well-demarcated hyperkeratotic, erythematous plaques affecting the palmar and plantar regions. It commonly affects individuals engaged in manual labor or agricultural work, where repeated friction and mechanical stress can aggravate the condition. Typical presentation includes thickened scaly plaques, painful fissures, burning, and restriction of hand and foot function. Unlike simple calluses, palmoplantar psoriasis tends to be recurrent and resistant to routine emollient therapy. Management includes regular emollients, keratolytic agents (urea or salicylic acid), topical corticosteroids, and vitamin D analogs. In resistant or severe cases, phototherapy or systemic therapy (such as methotrexate, cyclosporine, or acitretin) may be required. Identifying aggravating factors and providing long-term dermatological care are essential for effective management.

**Figure 1:**
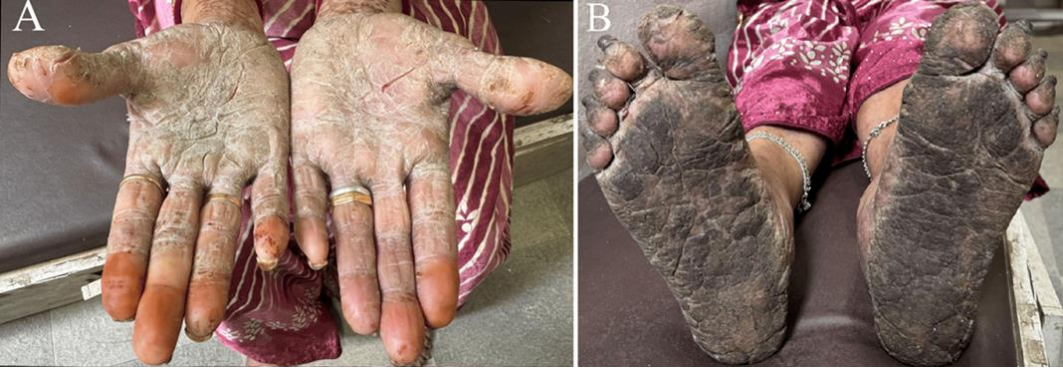
A) whitish scaling and deep fissures over the palm; B) blackish scaling and fissures over the sole region

